# Allergic airway inflammation induces upregulation of the expression of IL-23R by macrophages and not in CD3 + T cells and CD11c^+^F4/80^−^ dendritic cells of the lung

**DOI:** 10.1007/s00441-021-03538-0

**Published:** 2022-04-27

**Authors:** Maximilian Leitner, Sebastian Heck, Kenny Nguyen, Phu Quyen Nguyen, Shaza Harfoush, Eva Rosenkranz, Robert Bals, Quoc Thai Dinh

**Affiliations:** Department of Internal Medicine V, Saarland University Hospital, Homburg, Germany

**Keywords:** IL-23, IL-23R, Allergic airway inflammation, Macrophages, Airway neutrophilia

## Abstract

Interleukin 23 and the interleukin 23 receptor (IL-23-IL23R) are described as the major enhancing factors for Interleukin 17 (IL-17) in allergic airway inflammation. IL-17 is considered to induce neutrophilic inflammation in the lung, which is often observed in severe, steroid-resistant asthma-phenotypes. For that reason, understanding of IL-23 and IL-17 axis is very important for future therapy strategies, targeting neutrophil pathway of bronchial asthma.

This study aimed to investigate the distribution and expression of IL-23R under physiological and inflammatory conditions. Therefore, a house dust mite (HDM) model of allergic airway inflammation was performed by treating mice with HDM intranasally. Immunofluorescence staining with panel of antibodies was performed in lung tissues to examine the macrophage, dendritic cell, and T cell subpopulations. The allergic airway inflammation was quantified by histopathological analysis, ELISA measurements, and airway function.

HDM-treated mice exhibited a significant allergic airway inflammation including higher amounts of NE^+^ cells in lung parenchyma. We found only a small amount of IL-23R positives, out of total CD3^+^T cells, and no upregulation in HDM-treated animals. In contrast, the populations of F4/80^+^ macrophages and CD11c^+^F4/80^−^ dendritic cells (DCs) with IL-23R expression were found to be higher. But HDM treatment leads to a significant increase of IL-23R^+^ macrophages, only. IL-23R was expressed by every examined macrophage subpopulation, whereas only Mϕ1 and hybrids between Mϕ1 and Mϕ2 phenotype and not Mϕ2 were found to upregulate IL-23R. Co-localization of IL-23R and IL-17 was only observed in F4/80^+^ macrophages, suggesting F4/80^+^ macrophages express IL-23R along with IL-17 in lung tissue.

The study revealed that macrophages involving the IL-23 and IL-17 pathway may provide a potential interesting therapeutic target in neutrophilic bronchial asthma.

**Supplementary information:**

The online version contains supplementary material available at 10.1007/s00441-021-03538-0.

## Introduction

With about 300 million affected people worldwide, bronchial asthma is a very common disease (Braman 2006). The disease is characterized by reversible airway obstruction with various symptoms, such as wheezing, dry cough, dyspnea, airway hyperresponsiveness, and mucus hypersecretion (Patadia et al. 2014). Despite the fact that effective biologicals for the treatment of allergic and eosinophilic bronchial asthma are available, there is no effective targeted therapy for “neutrophilic” bronchial asthma.

Interleukin-23 is now considered to play a key role in asthma pathophysiology. Produced and secreted by antigen-presenting cells (APC), like macrophages and dendritic cells (DC) (Langrish et al. 2004), and more recently by eosinophil granulocytes (Guerra et al. 2017) and bronchial epithelial cells (Lee et al. 2017), it enhances the chronic inflammation. IL-23 is a heterodimeric protein, composed of p40, a subunit also known as a part of IL-12 and p19, which is specific for IL-23 (Oppmann et al. 2000). After release, it connects with the IL-23 receptor complex, a heterodimeric peptide as well, which is constructed of IL-12Rβ1 and IL-23R functioning as a JAK2 receptor. The binding of IL-23 at IL-23R leads into the activation of multiple signaling molecules, especially STAT3 (Parham et al. 2002).

Targets for IL-23 signaling are TH-17 cells, a T cell population accountable for IL-17 production. These T cells develop from naïve T cells under the influence of IL-6 and TGFβ in mice. In humans, the cytokine IL-1ß seems to adopt the effect of TGFβ (Veldhoen and Stockinger 2006; Acosta-Rodriguez et al. 2007). While IL-23 is not able to induce differentiation of TH17 cells, it has a key role for survival and IL-17 expression by this T cell subpopulation. This pathway is called the IL-23 and IL-17 axis (Iwakura and Ishigame 2006; Langrish et al. 2004; Sheibanie et al. 2007). The release of IL-17 leads to neutrophil recruitment, which is declared as a key point of pathogenesis in many chronic inflammatory diseases, like psoriasis, inflammatory bowel diseases, and bronchial asthma (Onishi and Gaffen 2010; Molet et al. 2001; He et al. 2007; Wilson et al. 2009). Especially, this IL-17-induced neutrophilia is often considered as a cause of steroid-resistant asthma (McKinley et al. 2008).

Despite the well-characterized interaction between IL-23 and TH17 cells and its role in chronic inflammatory diseases (Onishi and Gaffen 2010), there is little known about the interaction of IL-23 with other inflammatory cells. Another IL-23 responding cell population is the group of antigen-presenting cells, macrophages, and DCs. In a study performed by Wakashin et al., the release of IL-4 by CD4^+^ T cells was measured. DCs alone weren’t able to release significant levels. However, when IL-23 was added, the DCs started to release IL-4 (Wakashin et al. 2008). Furthermore, in a murine model for multiple sclerosis, the *autocrine loop* of IL-23 has been demonstrated to be responsible for its production, and IL-23 seems to stimulate the expression of the proinflammatory, APC-related cytokines IL-1β and TNF. A higher amount of IL-23R mRNA in CNS was found in inflammatory macrophages during autoimmune inflammation in the brain (Cua et al. 2003). Together, the role of IL-23 in chronic inflammatory diseases, such as asthma, leads to the assumption that this mediator would be a new target in asthma therapy. Furthermore, some examinations show that blocking IL-23 leads to an amelioration of inflammation correlating parameters in murine asthma (Lee et al. 2017; Guan et al. 2012).

Many studies reveal the importance of IL-23R expression. There are variants of the IL-23R gene, which are protective or, conversely, predisposing for asthma and other groups of IL-23R-related chronic inflammatory diseases (Abdollahi et al. 2016; Mosayebian et al. 2015). Under the influence of IL-6 and IL-23 itself, IL-23R expression seems to be upregulated (Yang et al. 2007; Che Mat et al. 2011, Ghoreschi et al. 2010). Several in vitro analyses revealed an increased level of total IL-23R mRNA in allergic airway inflammation (Peng et al. 2010; Durrant and Metzger 2010; Hamzaoui et al. 2014).

The present work aimed to investigate the expression of IL-23R by its targeting cells. In a HDM mouse model, the expression of IL-23R by T cells, macrophages, and DCs was evaluated to underline the impact of IL-23-IL-23R signaling as a potential target in neutrophilic allergic airway inflammation for a novel asthma therapy.

## Methods

### Animals

Eight to 10 female C57Bl6J mice were exposed with HDM extract (Greer Inc.) for 5 consecutive days over a total period of 7 weeks. To induce chronic allergic airway inflammation, we injected a diluent of 25 µg HDM in 50-µl PBS intranasally. For control animals, we injected 50 µl of pure saline via the same route. Animal experiments were performed in strict accordance with German animal protection law and approved by the appropriate governmental authority (No. 01/2014).

### Lung function testing

Lung function analysis was performed using a non-invasive technique with conscious animals. Therefore, we used a double-chamber head-out plethysmograph (DSI Buxco FinePointe NAM, MN, USA) to measure airway resistance (sRaw). After inserting the animals in the device, they were granted an acclimation period of 5 min. Testing the airway hyperresponsiveness was performed via enhancing of doses (0, 12.5, 25, and 50 mg/ml) of methacholine (MCh) via an aerosol nebulizer. Within 1 min, 0.02 ml of aerosol volume was delivered. Each MCh concentration was applied within an interval of 6 min, consisting of 3 -min response time and 3- min recovery period.

### Histological staining

We produced Zambonie fixed lung cryosections (8 μm), by using a cryostat (CM1950; Leica Cryostat, Germany). Our sections contained all lobes of the murine lungs in coronary cuts. To get an overview of the tissue and to quantify the allergic airway inflammation, the cryosections were stained with hematoxylin and eosin (H&E) and with periodic acid-Schiff (PAS) according to standard protocols (Table 1). Bronchial epithelium thickness and basement membrane length were measured using software AxioVision (Carl Zeiss).

**Table 1:** Protocols H&E and PAS staining

H&E	PAS
1. Drying slices at room temperature	1. Drying slices at room temperature
2. Incubation with hematoxylin	2. Rinsing with water
3. Cleaning with water	3. Incubation with periodic acid 0.1%
4. Incubation with eosin 0.5%	4. Cleaning with water
5. Cleaning with an ascending alcohol series	5. Incubation with Schiff reagent
6. Cleaning with xylol	6. Rinsing with water
	7. Incubation with hematoxylin
	8. Rinsing with water
	9. Cleaning with ammonia
	10. Cleaning with an ascending alcohol series
	11. Cleaning with xylol

For the measurement of epithelium thickness, HE-stained cryosections were used. We analyzed 13 to 15 only bronchioles per animal. Two contours were drawn, an outer contour around the basement membrane of the airway and an inner contour around the luminal pols of the epithelial cells. After the measurement of the area of both contours, the inner contour was subtracted of the outer contour. This difference was denoted as area of airway epithelial layer. The results of these measurements were expressed as mean of area (µm^2^) per 1-µm basement membrane. For the quantification of epithelial goblet cells, we used PAS-stained cryosections. With a minimum circumference of 500 µm, 7 to 8 bronchioles were measured. Intraepithelial goblet cells were counted, and a contour around the base membrane was drawn. Further, we divided the number of goblet cells by the circumferences of the base membranes. The results were expressed as mean of goblet cells per 1-mm basement membrane.

### Immunofluorescence staining

For immunofluorescence staining, we used Zambonie fixed lung cryosections (8 μm). Our sections contained all lobes of the murine lungs in coronary cuts. Cryosections of the lungs were stored at − 20 °C. Before staining, they were dried at room temperature for 15 min and then rehydrated in PBS for 5 min. The sections were put in a Shandon Sequenza system (Thermo Scientific, MA, USA) and washed twice with PBS to check the rate of flow through the cover plates. After that, we blocked the sections with a dilution of 5% donkey serum in PBS. To reduce non-specific bindings of the secondary antibodies, the host of the blocking serum conformed to the host of the secondary antibodies. The sections were then incubated with 150 µl per lung with primary antibodies (Table 2). After washing twice with PBS, the sections were incubated with secondary fluorescein-conjugated antibodies in a dark area. To simplify the identification of cells, the sections were then incubated with 80 µl DAPI (Carl Roth) with a dilution of 1 pg/ml in PBS, which is a typical marker for cell nuclei. After that, the sections were washed 3 times with PBS and once with double distilled water. Finally, we fixed them with 70 µl of fluorescent mounting medium FluoroShield (Sigma Aldrich), covered them with cover slips, and dried them for at least 5 h at room temperature.

**Table 2:** List of antibodies

Primary antibodies	Company	Dilution
Rat anti-mouse CD3	Biolegend, USA	1:100
Armenian Hamster monoclonal anti-mouse CD11c	Biolegend, USA	1:100
Rat monoclonal anti-mouse CD38	Acris, Deutschland	1:100
Rat monoclonal anti-CD 170 (Siglec-F)	eBioscience, USA	1:200
Goat polyclonal anti-c-Myc	Novus Biologicals, USA	1:200
Rabbit monoclonal anti-mouse F4/80	Abcam, UK	1:150
Rat monoclonal anti-mouse F4/80	eBioscience, USA	1:100
Rabbit polyclonal anti-IL17	Abcam, UK	1:200
Rabbit polyclonal anti-IL23 (p19)	Abcam, UK	1:100
Goat polyclonal anti-IL23 receptor-aminoterminal end	Abcam, UK	1:200
Rabbit polyclonal anti-IL23 receptor	Abcam, UK	1:150
Rabbit polyclonal anti-iNOS	Abcam, UKU	1:150
Rabbit polyclonal anti-neutrophil elastase	Abcam, UK	1:200
Rabbit polyclonal anti-RELM alpha	Abcam, UK	1:200
**Secondary antibodies**	**Company**	**Dilution**
Donkey anti-goat IgG cyanine Cy3	Jackson, USA	1:200
Donkey anti-rabbit IgG cyanine Cy3	Jackson, USA	1:300
Goat anti-Armenian hamster IgG cyanine Cy3	Jackson, USA	1:400
Donkey anti-rabbit IgG Alexa Fluor® 488	Jackson, USA	1:400
Donkey anti-rabbit IgG Alexa Fluor® 488-Fab fragment	Jackson, USA	1:200
Donkey anti-rat IgG Alexa Fluor® 488	Jackson, USA	1:400
Goat anti-Armenian hamster Dylight 649	Jackson, USA	1:100
Donkey anti-goat IgG Alexa Flour® 647	Jackson, USA	1:100
Donkey anti-rat IgG Alexa Flour® 647	Jackson, USA	1:200

Stainings with primary antibodies from the same host were performed on 3 days. On the first day, we added the first antibody, incubated overnight, and supplemented an appropriate Fab fragment antibody on the second day. After washing twice with PBS, we continued with the remaining primary antibodies like our earlier description.

### Immunofluorescence analysis

For the analysis of staining, epifluorescence microscope (HXP 120 V (Carl Zeiss)) was used, and the cell counting was done manually. We analyzed two, non-sequential, lung slices per animal. The analysis was compared with isotype controls, which were incubated with the secondary antibodies, only. Isotype controls and antibody-stained samples were compared with the same light exposure. To reduce the number of false-positive cells, only cells fluorescing brighter than isotype control were declared as positive (Supplementary Fig. 1).

The verification of a neutrophil influx was performed by immunofluorescence staining with neutrophil elastase (NE) which is a neutrophil-specific enzyme (Papayannopoulos et al. 2010). The quantity of neutrophil inflammation was expressed as number of NE^+^ cells per mm^2^ of lung parenchyma. To validate the amount of IL-23 producing cells, we performed triple immunofluorescence stainings with antibodies against IL-23, F4/80, and CD11c for the analysis of APC. IL-23 on eosinophils were analyzed using antibodies against IL-23, CD11c, and Siglec-F.

For quantification of IL-23 receptor on CD3^+^ cells, we performed double immunofluorescence staining. Analysis of IL-23 receptor expression on APC was performed by a triple immunofluorescence staining with IL-23-receptor, CD11c, and F4/80. For identification of Mϕ1 macrophages, the marker molecules iNOS and CD38 were used. To visualize Mϕ2 macrophages, we were using RELMα and c-Myc. The amounts of IL-23R^+^ cells were expressed as percentage of total cells and additionally as cells per mm^2^ of lung parenchyma. Afterwards, we performed a coexpression analysis of IL-23R and IL-17 and tried to identify coexpressing cells with antibodies against IL-17, CD11c, and F4/80.

### Lung homogenization

Tissue samples were stored at − 80 °C. Cryofixed samples of the lungs were defrosted and homogenized using a stirrer (Ultra Turrax). The lung homogenates were centrifuged, the supernatants were sampled, and the pellets were discarded. Total protein concentrations were measured using Pierce^TM^BCA Protein Assay Kit (Thermo Fisher), and we further adjusted the concentrations by diluting with PBS.

### Measurement of total IgE IL-23 and IL-17

The levels of serum-IgE were evaluated by using Mouse IgE Ready-Set-Go (eBioscience). The detection limit was 4 ng/ml and results were shown as ng/ml IgE. Concentration of IL-23 in LHG was measured with Mouse IL-23 DuoSet ELISA (R&D Systems). The limit of detection was 4 ng/ml and results were shown as ng/ml IL-23. Concentration of IL-17 in LHG was measured with Mouse IL-17 DuoSet ELISA (R&D Systems). The limit of detection was 4 ng/ml and results were shown as ng/ml IL-17.

### Statistical analysis

Countings were performed manually. Because of the obvious histopathological differences of both groups, no blinding was established. Data are shown as mean ± SEM. Statistical significance was surveyed by unpaired *t*-test using GraphPad Prism 4.03. Grubbs’ test was performed to detect significant outliers. *P* values < 0.05 were considered significant.

## Results

### HDM treatment induces increased airway hyperresponsiveness and allergic airway inflammation

**Fig. 1:**
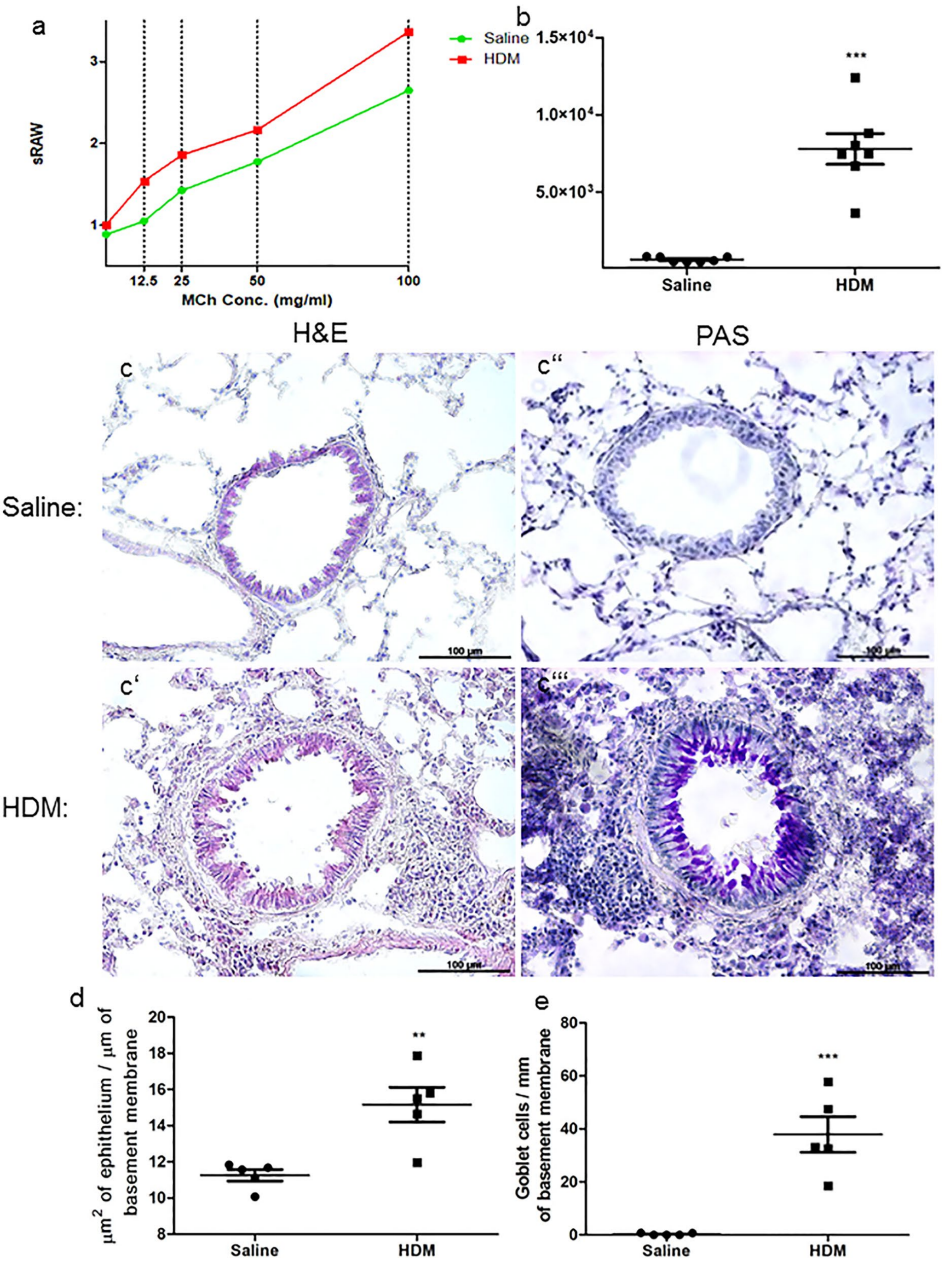
HDM treatment induces allergic airway inflammation. Allergic airway inflammation is characterized by increased airway hyperresponsiveness (**a**) and increased total serum IgE levels (**b**). Representative microphotographs of H&E- and PAS-stained lung cryosections (**c–c’’’**). H&E-stained sections of HDM-treated mice showed massive mononuclear infiltration, and PAS-stained sections demonstrated higher density of goblet cells and mucus within the bronchioles. In saline-treated controls, nearly no aggregated inflammatory cells, mucus, or goblet cells were found. Analysis of epithelium thickness per mm basement membrane (**d**) and number of goblet cells per 1-mm basement membrane (**e**). Results are expressed as mean ± SEM. **p < 0.01, ***p < 0.001 (unpaired two-tailed *t*-test). Scale bars: 100 µm

To confirm the effect of HDM-induced asthma on the airways, the airway hyperresponsiveness was objectified via MCh stimulation. Our results show that HDM-treated mice exhibited increased airway resistance replying to MCh in a dose-dependent manner (Fig. 1a). HDM treatment leads to a distinct rise of total serum IgE level (HDM 7792 ± 990.4 ng/ml *n* = 7 vs. saline 639.4 ± 61.65 ng/ml *n* = 7 *p* < 0.0001) (Fig. 1b). In comparison to controls, HDM-treated mice showed noticeably infiltration of mononuclear cells in H&E staining and increased goblet cell numbers in PAS staining. Further, histopathological analysis exhibited a significantly hypertrophy of airway epithelium (HDM 15.16 ± 0.9596µm^2^/µm *n* = 5 vs. saline 11.26 ± 0.3187µm^2^/µm *n* = 5 *p* = 0.0048) and goblet cell hyperplasia (HDM 37.91 ± 6.745 cells/mm *n* = 5 vs. saline 0.2865 ± 0.1759 cells/mm *n* = 5 *p* = 0.0005) after HDM treatment, compared with saline-treated controls (Fig. 1 c, d, e).

### IL-23 concentration and number of neutrophils are significantly increased in allergic airway inflammation

**Fig. 2:**
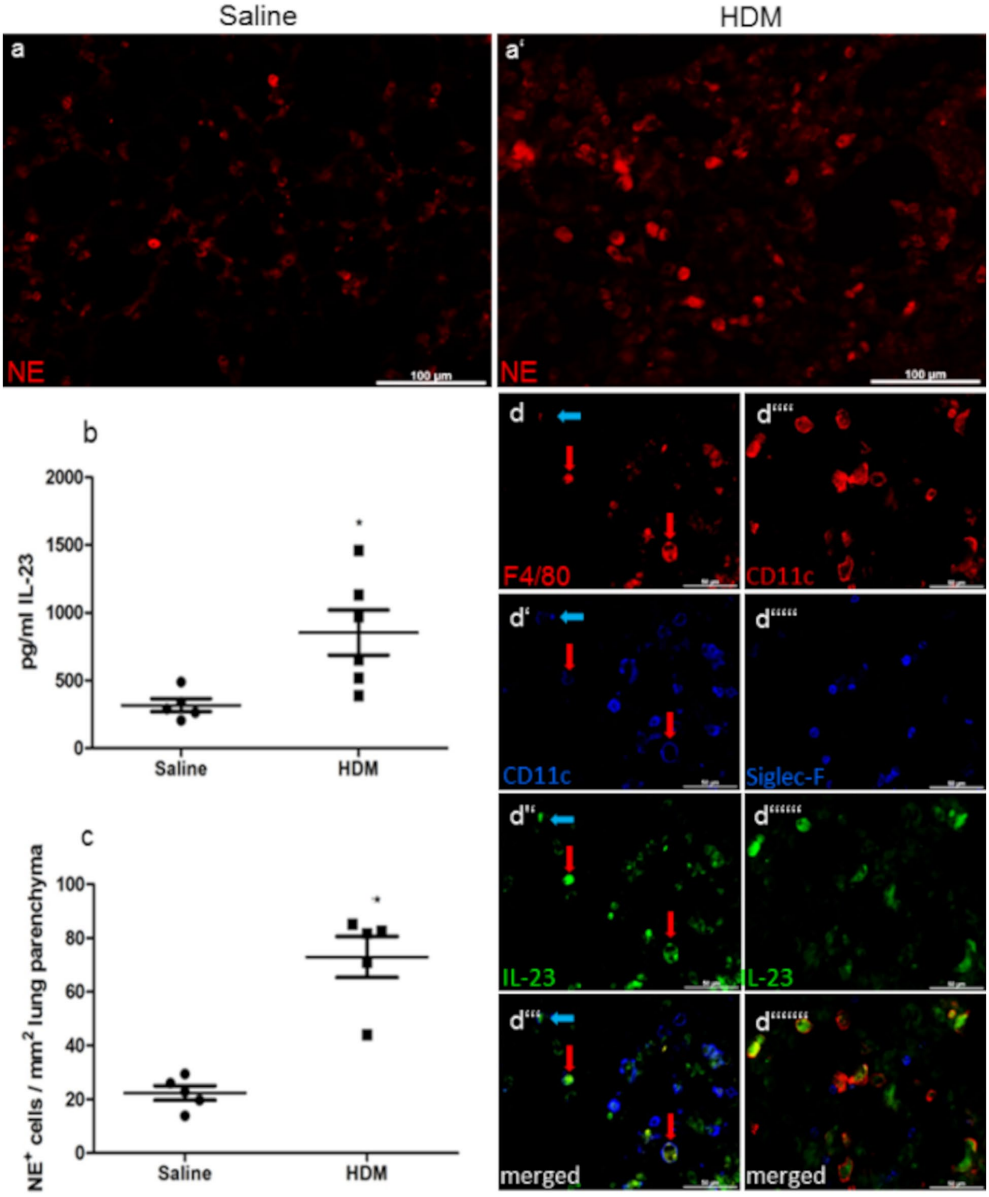
Number of neutrophils and IL-23 in lung tissue. Representative microphotographs of immunofluorescence staining with antibodies against NE indicate neutrophil infiltration into lung parenchyma (**a**). Quantification of IL-23 via ELISA of LHG showed a higher IL-23 concentration after HDM treatment (**b**; pg/ml IL-23). In comparison to controls, HDM-treated mice showed an increased value of NE^+^ cells per mm^2^ lung parenchyma (**c**). Representative microphotographs of immunofluorescence staining in HDM group to analyze which cells produce IL-23. We identified F4/80^+^ cells, presumably macrophages (red arrows) as the main IL-23 producing cells; some CD11c^+^F4/80^−^ cells, presumably dendritic cells (blue arrows), were found to be IL-23^+^ as well. Siglec-F^+^ CD11c^−^ cells, presumably eosinophils, did not show any IL-23 positivity (**d**). Results are expressed as mean ± SEM. *p < 0.05, ***p < 0.001 (unpaired two-tailed *t*-test). Scale bars: 100 µm in a and 50 µm in d

We were able to find higher concentrations of IL-23 in homogenates of the lung in HDM-treated mice (HDM 854.5 ± 166.40 pg/ml *n* = 6 vs. saline 318.3 ± 47.76 pg/ml *n* = 5, *p* = 0.0195) (Fig. 2c). In a following immunofluorescence analysis, the major IL-23^+^ cells were found to be F4/80 positives, presumably macrophages. Some DCs contained IL-23 as well, but we could not detect any IL-23-positive CD11c^−^Siglec-F^−^ eosinophils (Fig. 2d). As aforementioned, IL-23 is often considered to be responsible for the neutrophil component of autoimmune inflammation, by inducing IL-17 production (Aggarwal et al. 2003; Onishi and Gaffen 2010). For quantification of neutrophil granulocytes in lung parenchyma, cells containing neutrophil elastase (NE) were detected by immunofluorescence staining (Fig. 2a). Results were expressed as NE^+^ cells per mm^2^ of lung parenchyma. Under inflammatory conditions, the number of neutrophils in lung parenchyma was markedly increased if compared to saline-treated controls. (HDM 72.98 ± 7.614 *n* = 5 cells/mm^2^ vs. saline 22.36 ± 2.671 *n* = 5 *p* = 0.0002) (Fig. 2b).

### IL-23R is more frequently expressed at F4/80^+^ and CD11c^+^F4/80^−^ cells and then CD3^+^ cells; distinct increase after HDM treatment by F4/80 positives

**Fig. 3:**
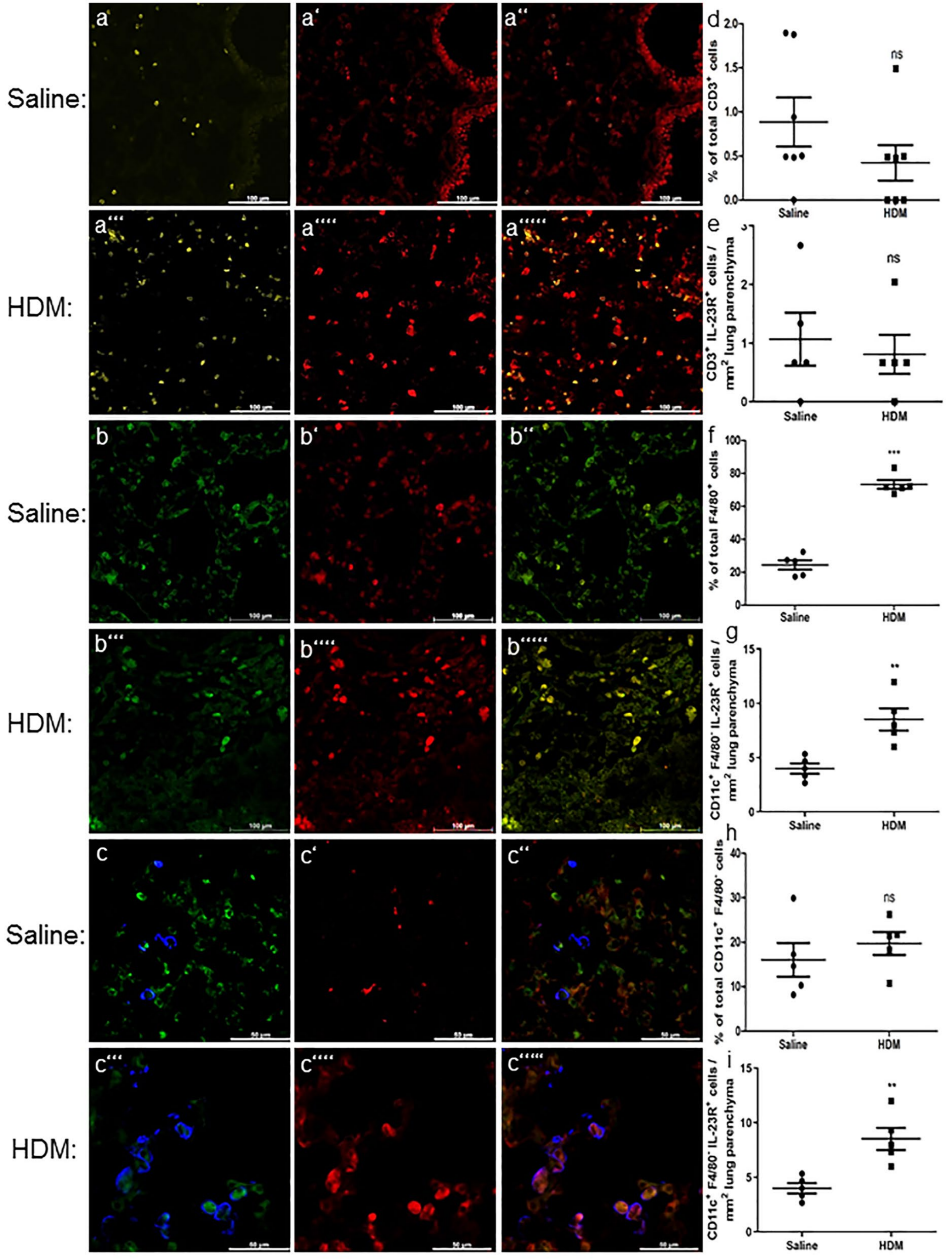
IL-23 receptor expression of different inflammatory cells. Representative microphotographs of immunofluorescence stainings with different inflammatory cell markers plus IL-23R. T lymphocytes were identified by CD3 expression (**a–a’’’’’**; CD3, yellow; IL-23R, red) and macrophages by F4/80 expression (**b–b’’’’’**; F4/80, green; IL-23R, red); CD11c-positive but F4/80-negative cells were declared as dendritic cells (**c–c’’’’’**; CD11c, blue; F4/80, green; IL-23R, red). CD3-positive cells exhibited only a very small rate of IL-23R positives and no significant change after HDM treatment (**d**). No significant increase of CD3^+^ IL-23R^+^ cells per mm^2^ lung parenchyma was observed (**e**). IL-23R was most frequently expressed by F4/80-positive cells. An increased rate of IL-23R positives of these cells in HDM-treated mice compared to saline-treated controls was observed (**f**). There was also a significant increase of F4/80^+^ IL-23R^+^ cells per area (**g**). CD11c^+^F4/80^−^ cells showed IL-23R positivity but no significant increase in IL-23 receptor positive rate under inflammatory conditions (**h**). In total cell count, we observed a significant increase of CD11c^+^ F4/80^−^ IL-23R^+^ cells per mm^2^ of lung parenchyma (**i**). Results are expressed as mean ± SEM. **p < 0.01, ***p < 0.001, ****p < 0.0001 (unpaired two-tailed *t*-test). Scale bars: 100 µm in a–a’’’’’and b–b’’’’’, 50 µm in c–c’’’’’

We first analyzed IL-23R expression of CD3^+^ cells. To evaluate the percentage of IL-23 receptor expressing CD3^+^ cells, different cryosections were stained with antibodies against CD3 and IL-23R (Fig. 3a). Surprisingly, only a very small amount of CD3^+^ cells were also viewed as IL-23R positive. No significant change under inflammatory conditions was detected. Thus, no differences could be observed either in the proportion (HDM 0.42 ± 0.20% *n* = 7 vs. saline 0.88 ± 0.28% *n* = 7 *p* = 0.20) or in the total cell count (HDM 0.81 ± 0.75 *n* = 5 vs. saline 1.01 ± 0.26 *n* = 5 *p* = 0.66) (Fig. 3d, e). Further, we were searching for IL-23R at other IL-23 targeting cells. A triple immunofluorescence staining with antibodies against IL-23R, F4/80, and CD11c was performed. Results were expressed as percentage of IL-23R-positive cells out of total F4/80^+^cells, percentage of IL-23R-positive cells out of total CD11c^+^F4/80^−^ cells, as well as cells per mm^2^ lung parenchyma (Fig. 3f, g, h, i). IL-23R was most frequently observed at F4/80^+^ cells and considerably increased in HDM-treated mice (HDM 82.38% ± 2.39% *n* = 5 vs. saline 40.31% ± 4.57% *n* = 5 *p* < 0.0001) (Fig. 3f). An increase in F4/80 + IL-23R + cells per area could also be shown (HDM 58.04 ± 6.6 *n* = 5 vs. saline 16.92 ± 4.44 *n* = 5 *p* < 0.0001) (Fig. 3g). CD11c^+^F4/80^−^ cells were found to be IL-23R positive as well, but less frequently as F4/80^+^ cells. Under inflammatory conditions, the amount of IL-23R positives was not significantly increased (HDM 19.73% ± 2.57% *n* = 5 vs. saline 16.05% ± 3.81% *n* = 5 *p* = 0.44) (Fig. 3h). However, there was an increase in total cell number of CD11c^+^F4/80^−^ IL-23R^+^ cells per area (HDM 8.53 ± 2.28 *n* = 5 vs. saline 4 ± 1.05 *n* = 5 *p* < 0.0038).

### Phenotyping of IL-23R-positive macrophages

**Fig. 4:**
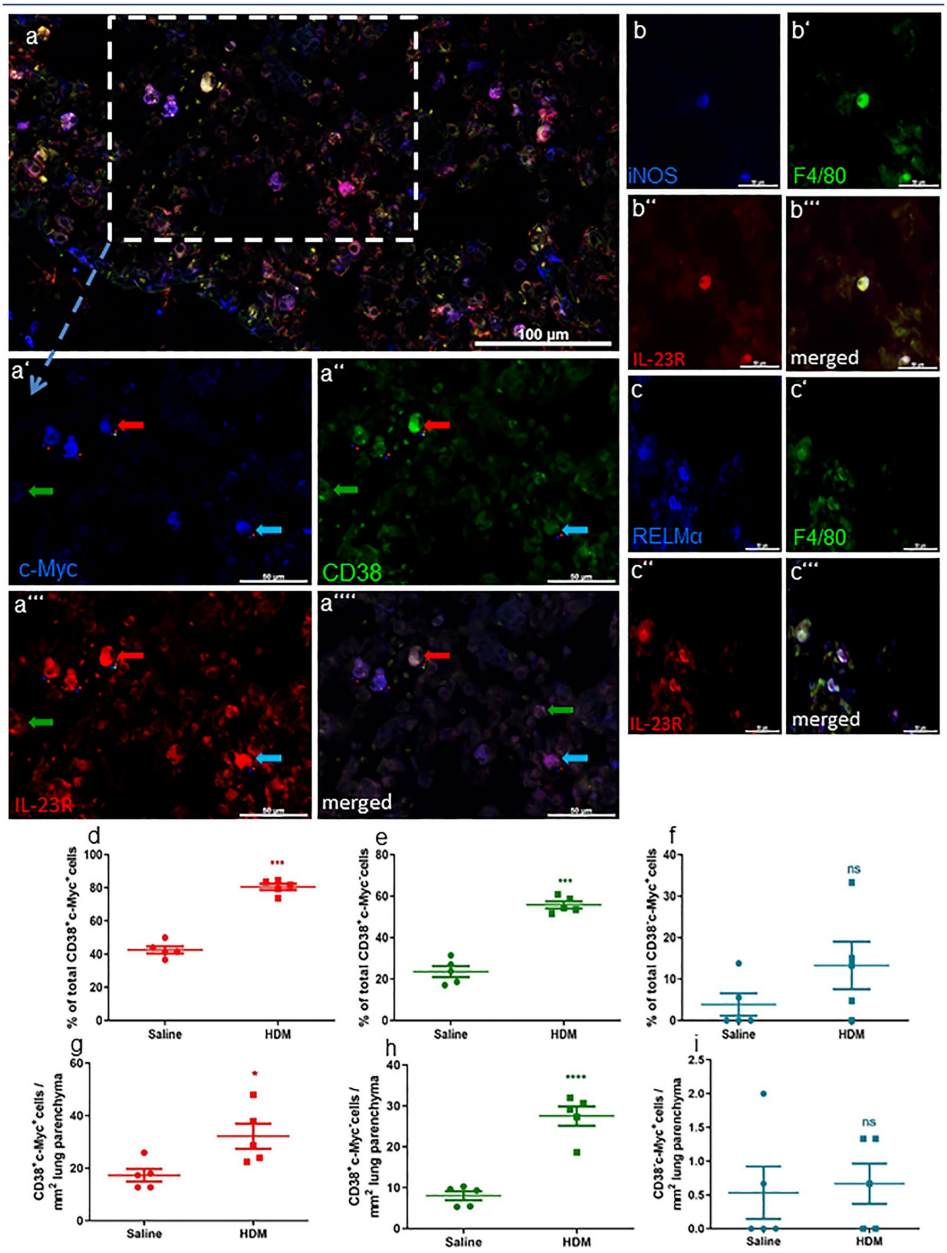
IL-23 receptor expression of macrophage subpopulations. Representative microphotographs of immunofluorescence staining with antibodies against c-Myc, CD38, and IL-23 receptor in HDM-treated mice. Red arrows: c-Myc^+^ CD38^+^ cells, presumably hybrids between Mϕ1 and Mϕ2. Green arrows: c-Myc^−^ CD38^+^ cells, presumably Mϕ1. Blue arrows: c-Myc^+^ CD38^−^ cells, presumably Mϕ2 (**a–a’’’’**). Microphotographs of fluorescence staining with iNOS (Mϕ1), F4/80 plus IL-23R (**b–b’’’**), and RELM alpha (Mϕ2) and F4/80 plus IL-23R (**c–c’’’**). c-Myc^+^ CD38^+^ cells showed the highest proportion of IL-23 receptor positives and a distinct increase after HDM treatment (**d**, **e**). c-Myc^+^ CD38^−^ cells showed IL-23R positivity and a distinct increase under inflammatory conditions, too (**f**, **g**), whereas only a few c-Myc^+^ CD38^−^ cells were positive for IL-23R and no significant rise in HDM-treated animals was detected (**h**,**i**). Results are expressed as mean ± SEM. ***p < 0.001 ****p < 0.0001 (unpaired two-tailed *t*-test). Scale bars: 100 µm in a, 50 µm in a’–a’’’’, b–b’’’, and c–c’’’

Based on our previous results, we further focused on IL-23R expression by macrophages as the main IL-23R-positive cell type. Therefore, we performed triple immunofluorescence stainings to differentiate between Mϕ1 and Mϕ2 phenotypes. At first, we detected co-localization of IL-23R, F4/80, and the Mϕ1 marker iNOS (Fig. 4b) as well as IL-23R, F4/80, and the Mϕ2 marker RELM alpha (Fig. 4c). Subsequently, the percentage of IL-23R positives per macrophage subpopulation was investigated by triple immunofluorescence staining with antibodies against IL-23R combined with the two novel macrophage phenotype markers CD38 and c-Myc (Jablonski et al. 2015) (Fig. 4a). IL-23 receptor was expressed by every macrophage phenotype. In HDM-treated mice, the percentage of IL-23R positives of CD38^+^c-Myc^+^ cells, presumably hybrids between Mϕ1 and Mϕ2 phenotypes, was increased (HDM 80.65 ± 1.92% *n* = 5 vs. saline 42.62 ± 2.19% *n* = 5 *p* < 0.0001) compared to saline-treated controls (HDM 80.65 ± 1.92% *n* = 5 vs. saline 42.62 ± 2.19% *n* = 5 *p* < 0.0001). The absolute cell number was also found to be significantly increased here (HDM 32.2 ± 10.7 *n* = 5 vs. saline 17.33 ± 5.4 *n* = 5 *p* = 0.0242) (Fig. 4d, e). CD38^+^c-Myc^−^ cells, presumably Mϕ1 macrophages, exhibited a higher percentage of IL-23 receptor positives under inflammatory conditions as well (HDM 55.76 ± 1.71% *n* = 5 vs. saline 23.58 ± 2.65% *n* = 5 *p* < 0.0001). The absolute cell number of IL-23R^+^ Mϕ1 macrophages was also found to be increased in HDM-treated mice (HDM 27.54 ± 5.26 *n* = 5 vs. saline 8.01 ± 2.41 *n* = 5 *p* < 0.0001) (Fig. 4f, g). No significant difference of c-Myc^+^CD38^−^ cells positive for IL-23R in HDM-treated animals compared to the controls was detected (HDM 13.29 ± 5.72% *n* = 5 vs. saline 3.87 ± 2.7% *n* = 5 *p* = 0.17) (HDM 0.67 ± 0.67 *n* = 5 vs. saline 0.53 ± 0.87 *n* = 5 *p* = 0.79) (Fig. 4f).

**Fig. 5:**
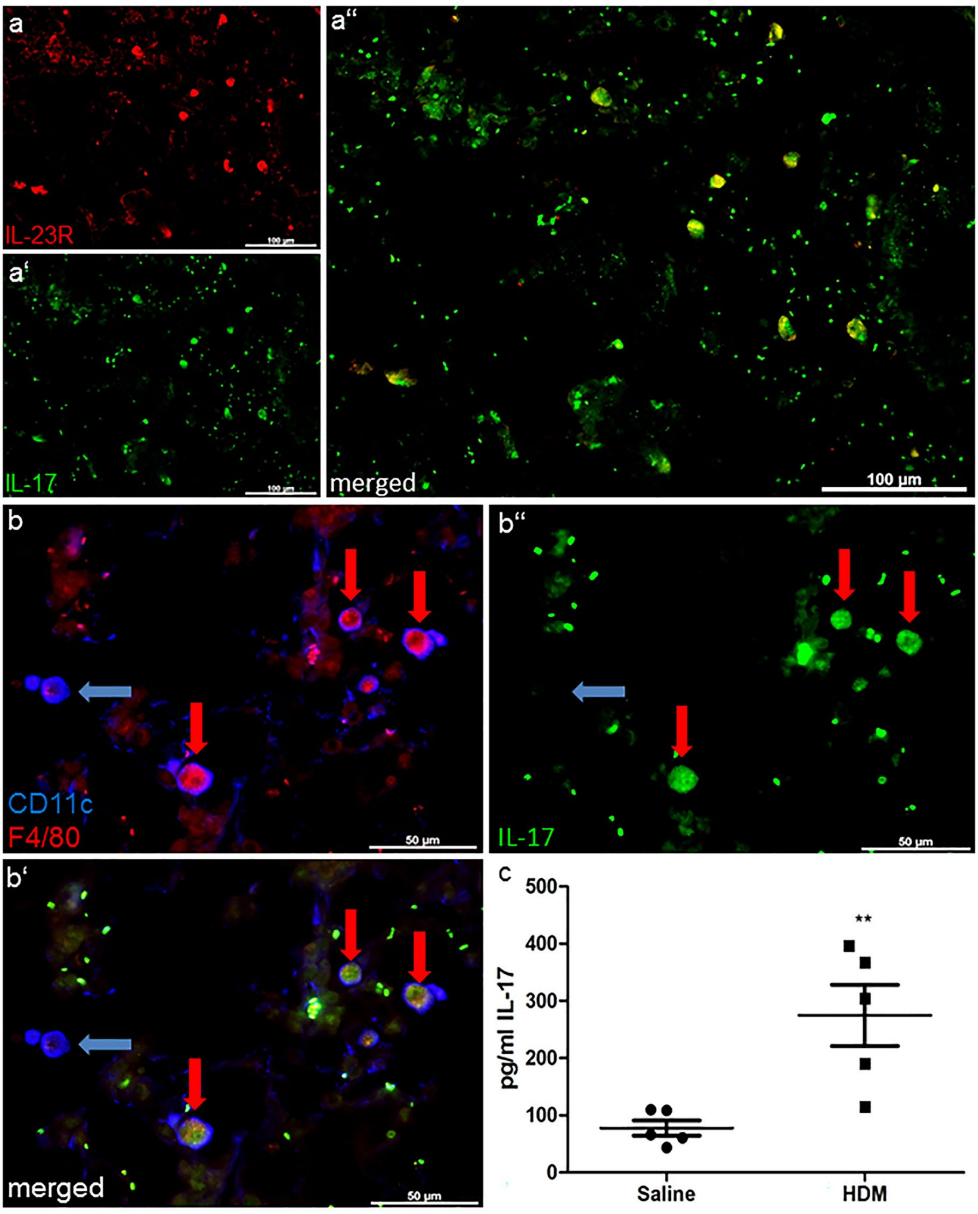
Co-localization of IL-17, IL-23 receptor, CD11c, and F4/80. Double immunofluorescence staining of IL-17 combined with IL-23R shows a distinct co-localization (**a–a’’**). Triple immunofluorescence staining of IL-17, F4/80, and CD11c showed co-localization of IL-17 and F4/80, but not of IL17 and CD11c^+^ F4/80^−^ (blue arrows, CD11c^+^ F4/80^−^ IL-17^−^; red arrows, CD11c^+^ F4/80^+^ IL17^+^) (**b–b’’**). HDM-treated mice exhibited higher IL-17 levels, compared to saline-treated controls (**c**). Results are expressed as mean ± SEM. **p < 0.01(unpaired two-tailed *t*-test). Scale bars: 100 µm in a–a’’, 50 µm in b–b’’

Furthermore, we explored the distribution of IL-23R and its effector cytokine IL-17. The concentration of IL-17 in homogenates of lung of HDM-treated mice was significantly increased, compared to saline-treated controls (HDM 274.6 ± 53.43 *n* = 5 vs. saline 77.70 ± 13.37 *n* = 5 *p* > 0.01) (Fig. 5c). In double immunofluorescence staining with antibodies against IL-23 receptor and IL-17, IL-17 and IL-23 receptor were very strictly co-localized (Fig. 5a). In triple immunofluorescence staining with the markers IL-17, CD11c, and F4/80, we figured out these cells were most likely F4/80^+^ (Fig. 5b) suggesting F4/80^+^ macrophages express IL-23R along with IL-17 in lung tissue.

## Discussion

HDM treatment leads to chronic airway inflammation, as we have proven, including significant neutrophil recruitment into airway parenchyma. Like Lambrecht and Hammad (2015) reported, although asthma is typically known for eosinophilia and increased TH2 cytokines, some patients, especially individuals with late-onset or more severe forms, manifest a neutrophil-derived inflammation as well (Lambrecht and Hammad 2015). For that reason, HDM mouse model turns out to be an interesting model for understanding the mechanisms of neutrophilia in asthma. Additionally, we could recognize higher concentrations of IL-23 in lung homogenate as well. IL-23 is regularly described as a cause of neutrophil infiltration in asthma (Li et al. 2011) and other inflammatory diseases (Dublin et al. 2012; Wang et al. 2015).

To our knowledge, this is the first work, demonstrating the distribution of IL-23R expression in a mouse model of allergic airway inflammation. At first, we exhibited just a very small percentage of IL23R positives out of total CD3^+^ cells. Although the TH17 cells, which are known as the main IL-23 targeting T cells (Wilson et al. 2007), are only a small population of total CD3^+^cells, their amount was observed to be increased under inflammatory conditions in the lung (Ming et al. 2016). But we were not able to notice higher percentages of IL-23^+^ CD3^+^ cells out of total CD3 positives, suggesting that most of these T cells express no or amounts of IL-23R below the detection limit.

In CD11c^+^ F4/80^−^ cells, presumably DCs, we found a higher percentage of IL-23R positives but no significant increase in HDM-treated mice. We believe that upregulation of IL-23R is a response to the higher IL-23 level under inflammatory conditions, as in different studies described (Che Mat et al. 2011; Ghoreschi et al. 2010). The fact that IL-23R was most frequently found at F4/80^+^ macrophages, and additionally, the heavy increase after HDM treatment reveals an impact of an autocrine IL-23-IL23R signaling. Another reason for this observation could be the increased influx of macrophages from the bloodstream. Other cytokines, like IL-6, could also have led to the upregulation of IL-23R. The information IL-23 brings to macrophages is still not completely understood and remains investigations in future studies. But the analysis of IL-23R expression of different macrophage phenotypes may allow a more differentiate look.

IL-23R was expressed most by c-Myc^+^CD38^+^ cells, followed by c-Myc^−^ CD38^+^, presumably Mϕ1 dominant macrophages, while c-Myc^+^ CD38^−^ cells, presumably Mϕ2 dominant macrophages, did only include a low percentage of IL-23R positives. In HDM-treated mice, the rate of IL-23R was only raised by c-Myc^+^ CD38^+^ and c-Myc^−^ CD38^+^ cell population but not by Myc^+^ CD38^−^ cells, which reveals the importance of these cells as IL-23 targeting cells. The significance of c-Myc^+^ CD38^+^ cells in the lung is very unclear and needs future research.

Alveolar Mϕ1 macrophages, which were characterized by c-Myc^−^ CD38^+^ phenotype (Jablonski et al. 2015), were often investigated in in vitro and in vivo studies of several inflammatory diseases (Liu et al. 2014). Interestingly, in murine models of other chronic inflammatory diseases, the amount of Mϕ1 macrophages correlates with the amount of TH17 cells, indicating Mϕ1 phenotype directly stabilizes TH17 phenotype (Geng et al. 2014; Li et al. 2012). Furthermore, in the group of macrophages, only Mϕ1 macrophages seem to produce IL-23 (Verreck et al. 2004). Together, this could imply the Mϕ1 part of macrophage initiated immunoreaction could be responsible for IL-17 production, probably via IL-23 release. Mϕ2 macrophages were characterized by c-Myc^+^ CD38^−^ phenotype (Jablonski et al. 2015), and only a few of them were found to be IL-23R positive. Combined with the fact that the rate of IL-23^+^ c-Myc^+^ CD38^−^ per c-Myc^+^ CD38^−^ cells was not significantly changed after HDM treatment, we thence hypothesize that IL-23-IL23R signaling plays a minor part in Mϕ2-derived inflammatory response.

Another study by Song et al. (2008) reveals that in allergic airway inflammation, IL-17 is not only produced by CD3^+^ T cells but also by CD11b^+^F4/80^+^ macrophages. We performed double Immunofluorescence staining with antibodies against IL-23R combined with antibodies against IL-17 and indeed recognized an almost entirely co-localization of these two markers. So, we hypothesize IL-23R^+^ macrophages additionally contain IL-17. In a subsequently triple immunofluorescence staining using antibodies against IL-17, F4/80, and CD11c, we could exhibit that these cells were F4/80^+^. Because of the previously described results, we assume that IL17^+^ IL-23^+^ cells were most likely macrophages. IL-17 was not detected on CD11c^+^ F4/80^−^ DCs. We think our in vivo findings confirm the hypothesis of IL-17 producing macrophages by the proof of IL-17^+^ F4/80^+^ cells and coexpression of IL-17 and its enhancing factor IL-23R in lung parenchyma.

## Conclusion

**Fig. 6:**
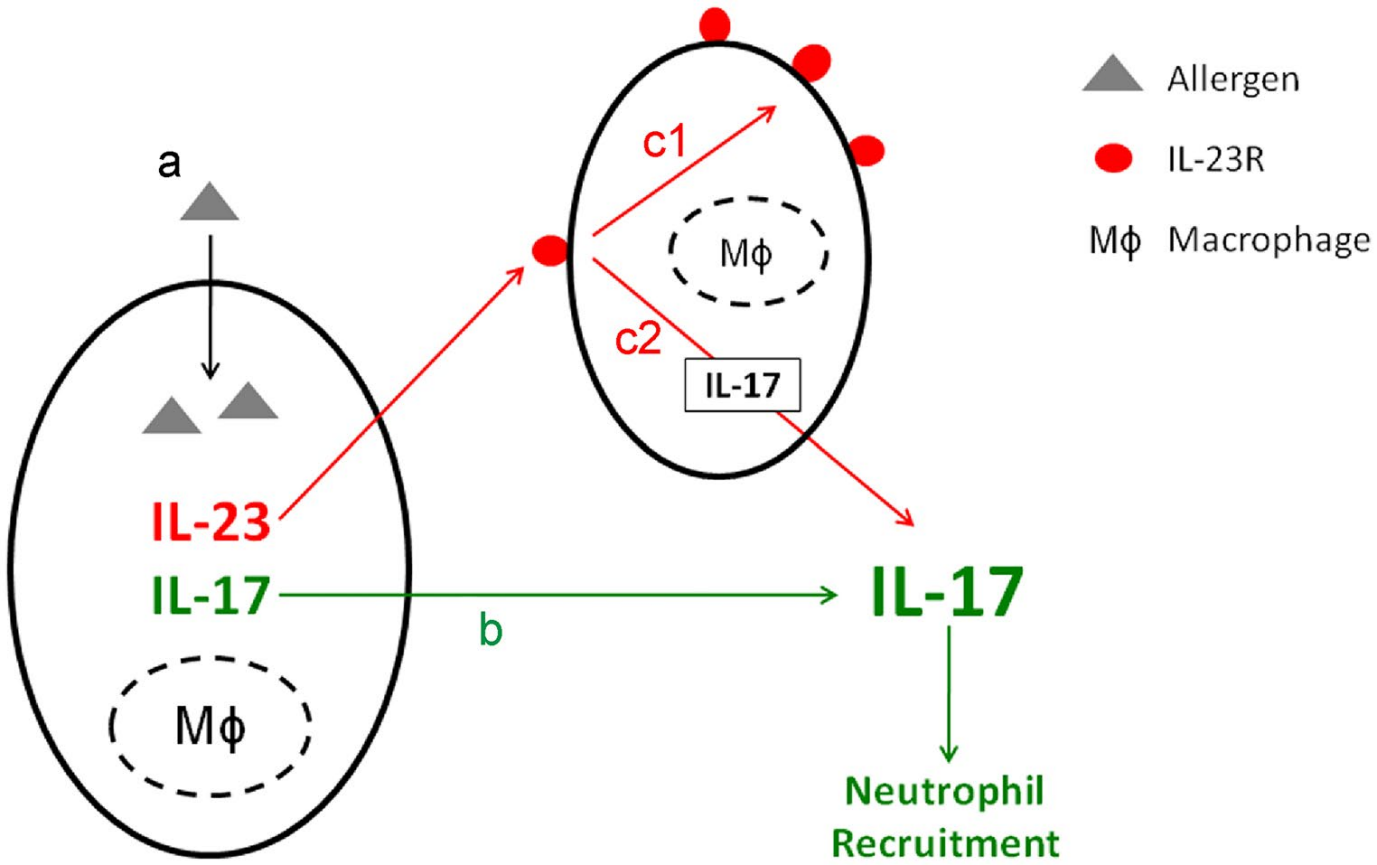
Theory of IL-23 and IL-17 signaling in bronchial asthma. Allergen uptake of macrophages (A). IL-17 response (green pathway): activation of Mϕ enhances IL-17 production, which causes neutrophil recruitment (B). IL-23 response (red pathway): activation of Mϕ leads to IL-23 secretion, which binds at IL-23R found on further, not yet activated Mϕ. IL-23-IL-23R signaling leads to two effects: the upregulation of IL-23R to ameliorate the later IL-23 reception (C1) and the IL-17 release to amplify the inflammatory response (C2)

The recent study demonstrates that one information IL-23 brings to macrophages could be the induction of IL-17 production via IL-23R pathway. We hypothesize IL-23 release could be kind of an “amplifier” in allergic response of the lung. After activation, local macrophages and DCs release IL-23 (Bosmann et al. 2013; Oriss et al. 2014) which activates other macrophages to enhance IL-17 productions which eventually provoke allergic airway inflammation, including neutrophil recruitment (He et al. 2007; Wilson et al. 2009) (Fig. 6).The co-localization of these two proteins in macrophages is nevertheless just a hint for the previously described mechanism. Future studies are necessary to further explore the very complex pathophysiology of IL-23 and IL-17 pathways for a potential interesting therapeutic target of a neutrophilic bronchial asthma.

## Supplementary information

Below is the link to the electronic supplementary material.

Supplementary file1 (DOCX 447 KB)
