# Supplementary material for: Allergic airway inflammation induces upregulation of the expression of IL-23R by macrophages and not in CD3 + T cells and CD11c+F4/80− dendritic cells of the lung
Source: Cell Tissue Res. 2022 Apr 27;389(1):85–98. doi: 10.1007/s00441-021-03538-0 (PMC9200692; doi:10.1007/s00441-021-03538-0)

**[Supplementary](sps:id::sec18) Fig. 1. Isotype vs. specific Antibody-staining**

Isotype and Donkey ant rabbit IgG Alexa Fluor® 488—Fab Fragment (A) vs. Rabbit monoclonal anti mouse F4/80 and Donkey ant rabbit IgG Alexa Fluor® 488—Fab Fragment (B). Isotype and Donkey anti rabbit IgG Cyanine Cy3 (C) vs. Rabbit polyclonal anti IL23 Receptor and Donkey**
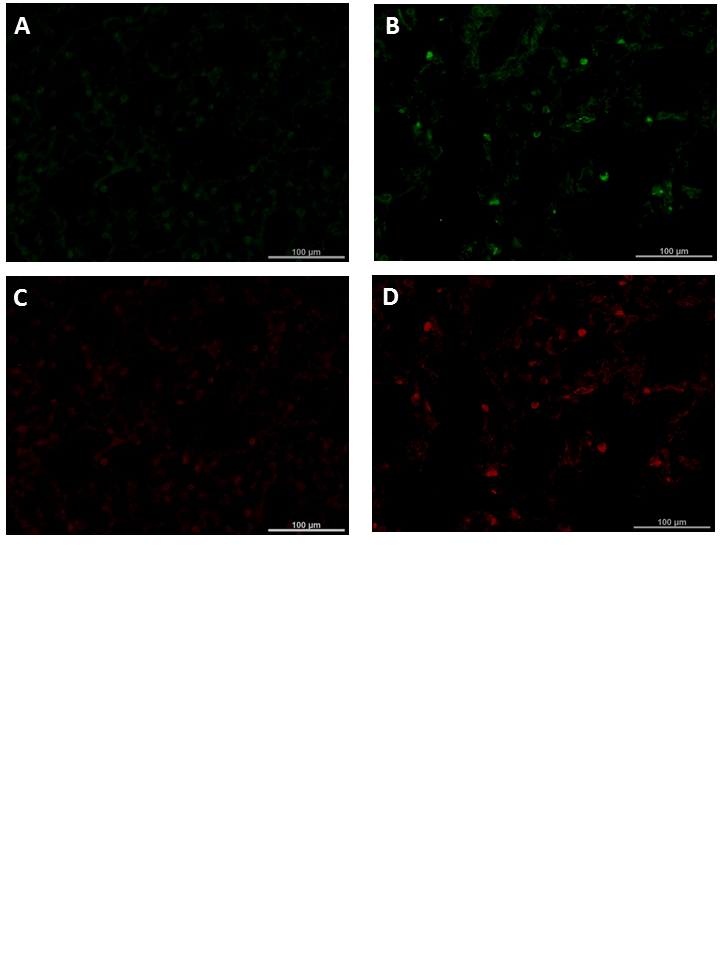
** anti rabbit IgG Cyanine Cy3 (D).


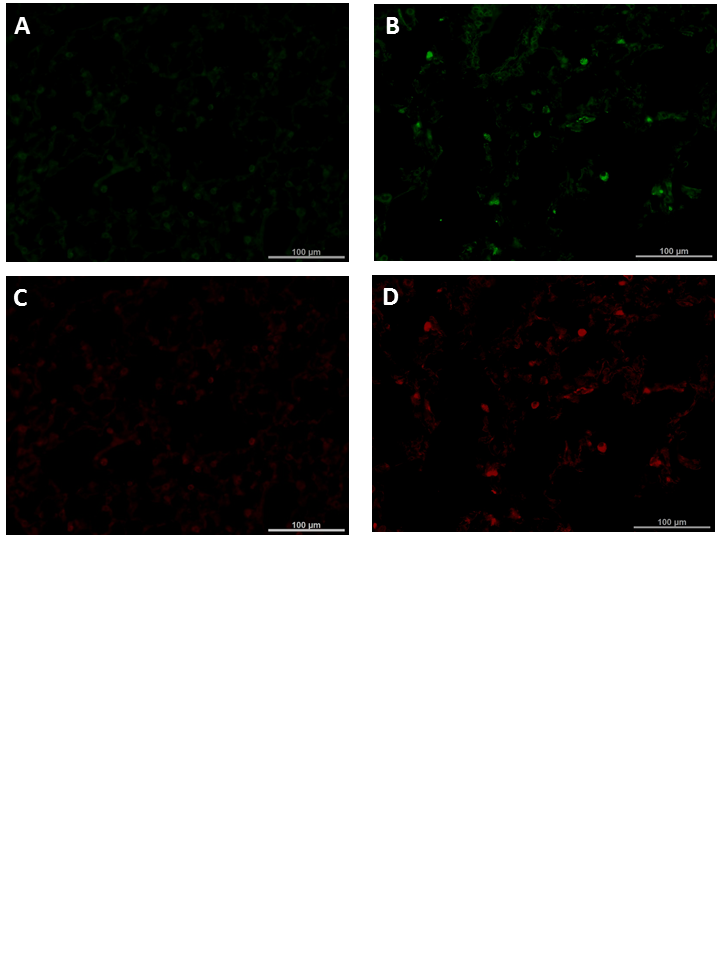

Supplement: Supplementary file 1 — Supplementary file1 (DOCX 447 KB) [file 441_2021_3538_MOESM1_ESM.docx]
